# Overweight dogs are more likely to display undesirable behaviours: results of a large online survey of dog owners in the UK

**DOI:** 10.1017/jns.2017.5

**Published:** 2017-04-26

**Authors:** Alexander J. German, Emily Blackwell, Mark Evans, Carri Westgarth

**Affiliations:** 1 Institute of Ageing and Chronic Disease, University of Liverpool, Neston, UK; 2 School of Clinical Veterinary Science, University of Bristol, Langford, UK; 3 Independent Veterinary Consultant, Guildford, UK; 4 Institute of Infection and Global Health, University of Liverpool, Liverpool, UK

**Keywords:** Welfare, Obesity, Canine nutrition, Anxiety, Aggression, Fearfulness

## Abstract

Much of the global canine population is now overweight, and this can adversely affect health, lifespan and quality of life. Undesirable behaviours are also common in pet dogs, and these can adversely affect welfare, as well as being stressful to owners. However, links between obesity and behavioural disorders have never previously been explored. An online survey was conducted between June and August in 2014, coinciding with the broadcast of a National UK television programme, exploring dog health, welfare and behaviour. Information gathered included signalment, overweight status and the prevalence of a range of undesirable behaviours. Fisher's exact test and OR were used to determine associations between overweight status and owner-reported behaviours. A total of 17 028 responses were received. After data verification, the final dataset comprised 11 154 dogs, 1801 (16·1 %) of which were reported by owners to be overweight. Owners of overweight dogs were more likely to see them as ‘a baby’ (*P* < 0·0001) and allow them to sleep on their bed (*P* < 0·0001). Overweight dogs were also more likely to guard food (*P* < 0·0001) and steal food (*P* < 0·0001). Other undesirable behaviours more commonly reported in overweight dogs included barking, growling or snapping at strangers (*P* = 0·0011) and other dogs (*P* = 0·0015), being fearful of outdoors (*P* < 0·0001), and not always coming back when called (*P* = 0·0011). Finally, owners were more likely to report that unsociable behaviours adversely affected their dog's health (*P* < 0·0001). Overweight status is associated with a number of undesirable behaviours in dogs. Further studies are now required to explore the reasons for these associations.

A multitude of problems can affect the welfare of dogs, ranging from inappropriate husbandry to euthanasia of healthy dogs. In a recent study, seven experts were asked to rank welfare priorities in dogs, and two of the top-rank priorities were undesirable behaviours and obesity^(^^1^^)^. Undesirable behaviours are known to be common in pet dogs, with one study estimating 90 % of the population to be affected^(^^2^^)^. Problems can include aggression to familiar people, strangers or other dogs; fearfulness; separation anxiety; and food-related behaviours such as food guarding, food stealing and coprophagia^(^^3^^)^. Obesity is also a considerable welfare concern in dogs; recent studies suggest that approximately half of all pet dogs are overweight^(^^4^^,^^5^^)^, and this can predispose dogs to many diseases^(^^4^^,^^6^^,^^7^^)^, poorer quality of life^(^^8^^)^ and a shorter lifespan^(^^9^^)^.

Whilst obesity and undesirable behaviours both have an impact on canine welfare, the degree to which they may be related is not known. Therefore, the aim of the present study was to examine the associations between overweight status and undesirable behaviours, based upon attitudes of owners in a large, anonymous, online survey.

## Methods and materials

### Study design and methodology

Over the summer of 2014, a television documentary series entitled ‘Dogs – Their Secret Lives’ aired on Channel 4 television. The four-part series dealt with the health and wellbeing of dogs in the UK, and aimed to inform the general public of issues of critical current importance. The series featured three of the study authors (A. J. G., E. B., M. E.). As part of the overall project, an online survey into the health and welfare of dogs was conducted between June and August in 2014, coinciding with the broadcast of a national UK television programme, exploring dog health, welfare and behaviour. The study was approved by the University of Liverpool Ethics Committee. Participation was voluntary, with owners wishing to participate logging onto the Channel 4 website in order to complete the survey. Owners gave their permission for their data to be used, in a fully anonymised form (i.e. no client-identifying data), and for the results to be publicised both on the television shows and online. Further, they were not required to answer questions that they were unclear about, or did not wish to answer. To be eligible for inclusion in the data analysis part of the study, dogs had to be adult (≥2 years of age) and questionnaire information needed to be complete, i.e. all questions used in the present study needed to be answered.

### Survey design

The online survey comprised forty-three questions, four of which covered personal data and these were not shared with the study investigators. Other questions covered details of signalment (age, sex, neuter status, breed), body weight and overweight status (based upon the response to the survey question: ‘Is your dog overweight?’), current body weight, lifestyle, questions on a range of undesirable behaviours, and activity. Free text boxes were available for the questions on age and body weight; the remaining questions were either binary in nature (yes/no) or categorical, and selections were made either by checking a box or selecting responses from a drop-down menu. Owners could only select one category. For overweight status, owners responded to the question, ‘Is your dog overweight?’, with their answer being based on their own subjective impression (i.e. no reference to a formal body condition scoring system). The main questions considered in the present study were those relating to undesirable behaviours, whereas the questions involving activity are reported in two associated studies^(^^10^^,^^11^^)^.

### Data handling and statistical analysis

All data were collated into a computer spreadsheet (Excel version 14; Microsoft) for analysis. Initially, data cleaning was conducted to ensure reliability of the data analysed. First, given that body weight was used to confirm overweight status (see below), it was necessary to ensure that growing dogs were not included in the dataset. This was done by removing data from all dogs under 2 years of age. The dataset was further cleaned by removing dogs with any missing data, and dogs with obvious errors in the dataset for age and body weight. The latter involved manually checking the spreadsheet for obvious data errors. First, the entire spreadsheet was inspected for obvious erroneous values (e.g. age or body weight entered as 0 or an improbable value, i.e. age >30 years, body weight >150 kg). Next, age data were sorted first by breed and then by age using the ‘sort’ function. For each breed, individual dog ages were checked and compared with expected age range of the age of the breed based upon that reported within an online encyclopaedia (https://www.wikipedia.org). Dogs with ages more than 20 % outside the range reported for each breed (given uncertainties of the reported age ranges) were removed. Finally, body weight data were sorted first by breed, then by sex, and then overweight status. Dogs that were reportedly not overweight but had a body weight more than 20 % above the range reported for each breed were removed.

Computer software (Stats Direct version 3.0.171; Stats Direct Ltd) was used for all tests. Data are expressed as median (range) unless otherwise stated. OR and 95 % CI were calculated in order to determine the association between owner-reported overweight status and various parameters, including sex, neuter status, and the responses to unsociable behaviours. Exceptions were for comparisons of age and body weight, where the Mann–Whitney test was used, and for the question ‘how do you see your dog?’, which had multiple categories and was assessed with Fisher's exact test. First, the test was performed across all categories simultaneously using the ‘r by c Fisher’ function in Stats Direct. Subsequently, the proportion of owners that selected the category ‘baby’ was compared with the proportion of owners selecting another answer using a 2 × 2 Fisher's exact test. Given that multiple comparisons were performed, a modified Bonferroni correction was applied^(^^12^^)^. This correction effectively meant that statistical significance was only considered when *P* < 0·0017.

## Results

### Final dataset

A total of 17 028 responses were received. After cleaning, the final dataset comprised 11 154 dogs, 6220 of which were male (4932 neutered; 79·3 %) and the remaining 4934 were female (4280 neutered; 86·8 %). Over eighty breeds were represented, with the most common being Border collie (583), Cavalier King Charles spaniel (224), cocker spaniel (512), mixed breed (2794), English springer spaniel (473), German shepherd dog (336), golden retriever (276), Jack Russell terrier (606), Labrador retriever (1344), Staffordshire bull terrier (451) and West Highland white terrier (217). Median age was 5 years (range 2–19 years) and median body weight was 20 kg (range 1–107 kg).

### Owner-reported overweight status

Of the 11 154 dogs, 1801 dogs were reported to be overweight (16·1 %). To confirm that the overweight population was representative, associations with various signalment variables were assessed. Overweight dogs were significantly older (6 (range 2–16) years *v.* 5 (range 2–19) years; *P* < 0·001) and heavier (22 (range 1–107) kg *v.* 20 (range 1–107) kg; *P* < 0·0001) than dogs that were reported to be of ideal weight. Overweight status was also positively associated with being neutered, but not with sex, whilst a range of breeds was either positively (beagle, Cavalier King Charles spaniel, Chihuahua, Labrador retriever and pug) or negatively (German shepherd dog and greyhound breeds) associated (Table 1).

**Table 1.** Association between signalment factors and overweight (OW) status (Number of dogs; odds ratios and 95 % confidence intervals)

Variable	OW	Not OW	OR	95 % CI	*P*†
Age (per year)	–	–	1·076	1·061, 1·092	<0·0001*
Sex					
Female (reference)	1583	8165	1·000	–	–
Male	218	1188	0·946	0·807, 1·06	0·509
Neuter status					
Entire (reference)	177	1765	1·000	–	–
Neutered	1624	7588	2·134	1·810, 2·19	<0·0001*
Body weight (per kg)	–	–	1·018	1·014, 1·022	<0·0001*
Breed					
Bassett	12	36	1·734	0·901, 3·343	0·099
Beagle	57	52	5·846	4·000, 8·543	<0·0001*
Bichon frise	14	60	1·213	0·677, 2·176	0·516
Border collie	74	509	0·744	0·580, 0·955	0·020
Border terrier	37	162	1·190	0·830, 1·707	0·345
Boxer	22	141	0·808	0·514, 1·270	0·355
Bull terrier	12	36	1·734	0·901, 3·343	0·099
Bulldog	14	30	2·435	1·288, 4·600	0·0061
Cairn terrier	14	41	1·779	0·968, 3·271	0·064
Cavalier King Charles spaniel	67	157	2·263	1·692, 3·026	<0·0001*
Chihuahua	29	63	2·413	1·550, 3·757	<0·0001*
Cocker spaniel	102	410	1·310	1·048, 1·637	0·018
Dachshund	20	75	1·389	0·846, 2·281	0·194
Dalmatian	5	66	0·392	0·158, 0·974	0·044
Doberman	9	51	0·916	0·450, 1·884	0·809
English springer spaniel	63	410	0·791	0·603, 1·036	0·088
Flat-coated retriever	3	46	0·338	0·105, 1·087	0·069
German shepherd dog	31	305	0·520	0·358, 0·754	0·0006*
Golden retriever	62	214	1·522	1·142, 2·029	0·0041
Greyhound	9	182	0·253	0·129, 0·495	<0·0001*
Hungarian Vizla	4	51	0·406	0·146, 1·125	0·083
Irish setter	2	38	0·272	0·066, 1·131	0·073
Jack Russell terrier	99	507	1·015	0·813, 1·266	0·896
Labrador retriever	285	1059	1·472	1·278, 1·697	<0·0001*
Lhasa apso	17	53	1·672	0·966, 2·894	0·066
Miniature Schnauzer	25	95	1·372	0·880, 2·137	0·162
Poodle	3	76	0·204	0·064, 0·646	0·0069
Pug	17	22	4·042	2·142, 7·626	<0·0001*
Rhodesian ridgeback	7	37	0·982	0·437, 2·207	0·966
Rottweiler	18	86	1·088	0·653, 1·812	0·746
Rough-coated collie	11	53	1·078	0·562, 2·068	0·820
Shih Tzu	19	83	1·191	0·721, 1·965	0·494
Siberian husky	7	61	0·594	0·271, 1·301	0·193
Staffordshire bull terrier	85	366	1·212	0·955, 1·549	0·112
Weimaraner	5	47	0·551	0·219, 1·388	0·206
West Highland white terrier	41	176	1·215	0·861, 1·713	0·267
Whippet	5	68	0·380	0·153, 0·944	0·037
Yorkshire terrier	19	96	1·028	0·627, 1·687	0·912

* Results reaching statistical significance.

† Modified Bonferroni correction applied, so statistical significance considered when *P* < 0·0017.

### Associations between owner attitudes and overweight status

When owners were asked how they viewed their dog, those that owned overweight dogs were more likely to select ‘your baby’ (507/2548 (20 %) overweight; OR 1·538; *P* < 0·0001) than to use one of the other descriptions (total 1294/8606 (15 %) overweight: ‘your protection’, 1/35 (3 %); ‘your assistant’, 3/36 (8 %); ‘your companion’, 570/3812 (15 %); ‘your best friend’, 339/2089 (16 %) or ‘your pet’, 381/2634 (14 %)). Owners of overweight dogs were also more likely to allow them to sleep in or on their bed (OR 1·432; 95 % CI 1·293, 1·539; *P* < 0·0001), but not more likely to keep a photograph of their pet with them (OR 1·034; 95 % CI 0·927, 1·153; *P* = 0·5522).

### Associations between overweight status and undesirable behaviours

Associations between overweight status and a range of undesirable behaviours were tested (Table 2). Dogs that were reported to be overweight were more likely to guard food (*P* < 0·0001), steal food (*P* < 0·0001), not always come back when called (*P* = 0·0002), be fearful or reluctant to walk outside (*P* < 0·0001), and be more likely to bark/growl/snap at other dogs (*P* = 0·0015) and strangers (*P* = 0·0011). Dogs that did not always come back when called were less likely to be let off the lead than those that always returned (OR 0·425; 95 % CI 0·388, 0·465; *P* < 0·0001). Similar associations were also seen between dogs barking, growling at both other dogs (OR 0·410; 95 % CI 0·374, 0·449; *P* < 0·0001) and strangers (OR 0·650; 95 % CI 0·581, 0·727; *P* < 0·0001) and the likelihood of being let off the lead. However, a direct association between overweight status and being let off the lead was not identified (OR 0·908; 95 % CI 0·807, 1·023; *P* = 0·1060). Further, there was no association with a range of other behaviours (Table 2).

**Table 2.** Association between behaviours and overweight status (Proportion and percentage of overweight dogs in each response category; odds ratios and 95 % confidence intervals)

	Yes		No				
Which of the following behaviours does your dog display on a regular basis?	*n*	%	*n*	%	OR	95 % CI	*P*†
Eat faeces	364/1993	18·3	1437/9161	15·7	1·201	1·055, 1·365	0·0053
Guard food items/possessions	261/1151	22·7	1540/10003	15·4	1·612	1·384, 1·872	<0·0001*
Steal food or scavenge items	617/2765	22·3	1184/8389	14·1	1·748	1·565, 1·951	<0·0001*
Bark, howl, destroy, toilet or chew things at home	174/1148	15·2	1627/10006	16·3	0·920	0·772, 1·092	0·3517
Toilet indoors when home alone	62/334	17·6	1739/10820	16·1	1·190	0·884, 1·582	0·2270
Bark/whine when not home alone	305/1733	17·6	1496/9421	15·9	1·131	0·985, 1·297	0·0758
Avoid/run away from							
Other people	148/874	16·9	1653/10280	16·1	1·064	0·879, 1·282	0·5029
Other dogs	233/1378	16·9	1568/9776	16·0	1·065	0·912, 1·240	0·4117
Not always come back when called	842/4771	17·6	959/6383	15·0	1·212	1·093, 1·343	0·0002*
Pull on lead	869/5534	15·4	932/5520	16·9	0·898	0·810, 0·994	0·0371
Jump up or try to get attention from people	798/5086	15·7	1003/6068	16·5	0·940	0·848, 1·041	0·2348
Bark growl snap at							
Other dogs	629/3535	17·8	1172/7619	15·4	1·191	1·069, 1·326	0·0015*
Strangers	346/1847	18·7	1455/9307	15·6	1·244	1·089, 1·418	0·0011*
Familiar people	114/536	21·3	1687/10618	15·9	1·344	1·076, 1·668	0·0078
Chase							
Traffic	91/523	17·4	1710/10631	16·1	1·099	0·862, 1·389	0·4288
Other things like cyclists, joggers	188/1057	17·8	1613/10097	16·0	1·138	0·958, 1·346	0·1352
Fearful of noises – run away, hide, whimper	188/1057	17·8	1613/10097	16·0	1·018	0·896, 1·156	0·7726
Fearful of outside – reluctant to walk	65/254	25·6	1736/9484	15·9	1·815	1·341, 2·431	<0·0001*
Obsessively lick himself/herself or people	304/1670	18·2	1497/9484	15·8	1·187	1·0328, 1·362	0·0142
Spin or chase his/her tail	109/857	12·7	1692/10297	16·4	0·741	0·596, 0·914	0·0043
Have the selected behaviours affected your dog's health or happiness?	752/4099	18·3	1049/7055	14·9	1·286	1·159, 1·427	<0·0001*

* Results reaching statistical significance.

† Modified Bonferroni correction applied, so statistical significance considered when *P* < 0·0017.

### Influence of undesirable behaviour and health

More owners of overweight dogs reported that behavioural issues adversely affected health (752/1801, 42 %) than for owners who did not believe their dog to be overweight (3347/9353, 36 %; OR 1·286; 95 % CI 1·159, 1·427; *P* < 0·0001).

## Discussion

This study describes the largest survey of owner attitudes to canine behaviour and welfare ever conducted, and some intriguing associations were identified between obesity and undesirable behaviour, including for the food-related behaviours (guarding and stealing food), displays of aggression to strangers or other dogs, being fearful of walking, and not returning when called. Furthermore, owners of overweight dogs were more likely to report that such undesirable behaviours were more likely to affect health than did owners of dogs not reported to be overweight. Given the fact that the study was cross-sectional in nature, the reasons for these associations cannot be determined, including whether or not they are causal. Prospective studies should now be considered to explore the nature of these associations more completely.

Assuming that the associations discovered are genuine, the link between overweight status and both food-seeking and food-guarding behaviour is perhaps most logical. For example, there might be common risk factors for both, for example that cause a stronger drive for food, making affected dogs more likely both to display food-related behaviours and to overeat causing weight gain. Such factors could be genetic and, in this regard, a deletion in the canine pro-opiomelanocortin gene was discovered in some dogs of the Labrador retriever breed, and this was found to be associated with both appetite and weight status in affected dogs^(^^13^^)^. Alternatively, the behaviour of the owner might be important, whereby different styles of ownership might mean different feeding regimens, food types and rewards, predisposing some dogs to weight gain and undesirable food-related behaviour. Here, a parallel can be drawn with parenting and childhood obesity. In this respect, different parenting styles have been identified which reflect differences in the degree of control the parent places over their child's behaviour and the responsiveness of the parent to their child's wishes^(^^14^^)^. The children of parents who display either an indulgent style (i.e. displaying warmth and respect for their child's needs but only limited monitoring of their behaviour) or an authoritarian style (i.e. making high demands on their children whilst showing little responsiveness to their opinions or wishes) are more likely to be overweight that those who show other styles^(^^14^^,^^15^^)^. There are many parallels between how parents care for children and how owners care for their dogs, and a recent review considered how pet ownership styles could be mapped to parenting styles^(^^16^^)^. If dog-ownership styles exist that are similar to the indulgent and authoritarian parent styles, then similar predispositions might exist for canine obesity. Whilst the present work did not examine this directly, it was notable that owners who reported their dog to be overweight appeared to have a different relationship with them, in that they were more likely to see them as a baby and more likely to let them sleep in or on the bed. Further, other studies have demonstrated that owners of overweight dogs feed them more snacks and table scraps, observe them more closely during feeding and also more often allow the dog to be present when preparing a meal^(^^17^^,^^18^^)^. Additional studies could examine attitudes and opinions of owners towards feeding their dogs, and explore the similarity to parenting behaviour more fully.

Possible reasons for associations between being overweight and other undesirable behaviours are less clear. It is possible that owners whose dogs demonstrate aggressive behaviours to strangers and other dogs are less likely to exercise them outside or restrict their freedom if they do^(^^19^^)^. Indeed, we found that such dogs were less likely to be let off the lead than those not displaying such behaviours. However, given that there was no direct association between dogs being let off the lead and overweight status, other factors might also contribute. One alternative reason for the link between undesirable behaviours and overweight status may be the incorrect practice of reward-based behaviour therapy/dog training, in terms of excessive food rewards to reinforce positive behaviour, thereby encouraging overeating. For this reason, owners should always be recommended to adjust the dog's food intake appropriately and ideally use the pre-measured daily food ration for training.

One final observation was the fact that owners of overweight dogs were more likely to state that undesirable behaviours adversely affected their dog's health. The reason for this association is not clear. It might be a direct association, for example the overweight status making the behaviour more severe. Alternatively, the association might represent a cumulative effect on health. In this respect, obesity is known to adversely affect quality of life in dogs^(^^8^^)^, so it might appear that dogs also displaying an undesirable behaviour are perceived to have a greater adverse effect on health simply because the dog is starting from a lower baseline quality of life. Once again, further studies are required both to confirm this association and to explore the reasons for it.

The study has a number of limitations that should be considered. Most notable was the fact that only 16·1 % of dogs were reported to be overweight by their owner, which is considerably less than current estimates of overweight and obesity in UK dogs^(^^5^^)^. The reason for this is not clear. One explanation might be participation bias, whereby the owners who were willing to participate were not representative of the UK dog-owning population. For example, dog owners could have been reluctant to participate if they knew their dog was overweight or were concerned. A second possible explanation for the low prevalence of overweight dogs might be misinterpretation of body shape by owners, since recent studies have demonstrated that owners underestimate the true body condition of their dog, often not realising they are overweight^(^^20^^)^. This is a concern for the present study because owners were not given any criteria to use for deciding whether their dog was overweight. The upshot of this would be many overweight dogs being inappropriately classified as not overweight, and this would tend to obscure differences between groups. Therefore, the true effect size of the associations between overweight status and undesirable behaviours might actually be stronger than was seen in the current study. Ideally, therefore, the current findings should now be confirmed with studies using different methods of assessing overweight status, for example the use of body condition scoring conducted by a trained veterinary professional.

A second limitation was the fact that the use of an online format meant that response options were limited, with owners having to choose from categories or binary options (i.e. yes or no). Therefore, there was no opportunity to explore the observations in any more detail, and the fact that the survey was fully anonymised means that further information cannot be obtained from the owners who participated. Third, given that the survey was conducted in association with a television documentary series examining behaviour and obesity in dogs, results might have been influenced by response bias, with the effect that owners consciously or subconsciously adapted their responses having watched the programmes.

Finally, this study is limited by the fact that it is exploratory in nature, and only examined simple associations without adjustment for confounding. This was not undertaken because of the multiple comparisons that had already been performed. It is likely that behavioural variables may correlate with confounders such as reduced exercise, or breed, which also lead to overweight. Future studies purposefully designed to examine independent associations should use multivariable analyses to adjust for confounding variables.

### Conclusions

In the present study, overweight status in dogs (as reported by their owners) was associated with a number of undesirable behaviours including food stealing, food guarding and aggression both to other dogs and strangers. Further studies are now required to explore the reasons for these associations.
